# Intraoperative Scintigraphy Using a Large Field-of-View Portable Gamma Camera for Primary Hyperparathyroidism: Initial Experience

**DOI:** 10.1155/2015/930575

**Published:** 2015-01-06

**Authors:** Nathan C. Hall, Robert L. Plews, Amit Agrawal, Stephen P. Povoski, Chadwick L. Wright, Jun Zhang, Edward W. Martin, John Phay

**Affiliations:** ^1^Department of Radiology, The Ohio State University Wexner Medical Center, 395 West 12th Avenue, 4th Floor, Columbus, OH 43210, USA; ^2^Department of Surgery, The Ohio State University Wexner Medical Center, 410 West Tenth Avenue, Columbus, OH 43210, USA; ^3^Department of Otolaryngology, The Ohio State University Wexner Medical Center, 410 West Tenth Avenue, Columbus, OH 43210, USA

## Abstract

*Background.* We investigated a novel technique, intraoperative ^99 m^Tc-Sestamibi (MIBI) imaging (neck and excised specimen (ES)), using a large field-of-view portable gamma camera (LFOVGC), for expediting confirmation of MIBI-avid parathyroid adenoma removal. *Methods.* Twenty patients with MIBI-avid parathyroid adenomas were preoperatively administered MIBI and intraoperatively imaged prior to incision (neck) and immediately following resection (neck and/or ES). Preoperative and intraoperative serum parathyroid hormone monitoring (IOPTH) and pathology (path) were also performed. *Results.* MIBI neck activity was absent and specimen activity was present in 13/20 with imaging after initial ES removal. In the remaining 7/20 cases, residual neck activity and/or absent ES activity prompted excision of additional tissue, ultimately leading to complete hyperfunctioning tissue excision. Postexcision LFOVGC ES imaging confirmed parathyroid adenoma resection 100% when postresection imaging qualitatively had activity (ES) and/or no activity (neck). The mean ± SEM time saving using intraoperative LFOVGC data to confirm resection versus first IOPTH or path result would have been 22.0 ± 2 minutes (specimen imaging) and 26.0 ± 3 minutes (neck imaging). *Conclusion.* Utilization of a novel real-time intraoperative LFOVGC imaging approach can provide confirmation of MIBI-avid parathyroid adenoma removal appreciably faster than IOPTH and/or path and may provide a valuable adjunct to parathyroid surgery.

## 1. Introduction

Primary hyperparathyroidism (PHPT) is a benign, but potentially debilitating, disease where hyperfunctioning parathyroid adenoma(s) can lead to elevated parathyroid hormone (PTH) levels with resultant hypercalcemia and associated morbidities [1]. In the majority of cases, PHPT is caused by a single hyperfunctioning parathyroid adenoma, with multigland disease occurring ≤1% of the time [2]. Thus, the standard of care for patients presenting with PHPT is localization and surgical resection of the offending adenoma [3–5]. Historically, surgery involved a bilateral neck exploration, visualization of all four parathyroid glands, and excision of any grossly abnormal glands. Adequate resection was indicated by normalization of serum PTH levels and pathologic confirmation of the specimen [6]. This method yielded a 95% success rate when performed by experienced surgeons [5, 7, 8].

The advent of MIBI imaging for preoperative identification of parathyroid adenomas allowed surgeons to accurately localize parathyroid pathology with preoperative diagnostic imaging and use intraoperative handheld gamma detection probes (HGDP) to assist in intraoperative localization and resection of abnormal parathyroid tissue if MIBI was injected on the same day surgery. Minimally invasive radioguided parathyroidectomy (MIRP) utilizes a small 3 cm unilateral neck incision, as opposed to the traditional “collar” incision for 4-gland investigation [7, 9–21]. The technique for utilizing MIBI and a HGDP was first described by our group in 1995 [22]. Furthermore, the addition of intraoperative ultrasound to the HGDP further improved the sensitivity and specificity for determining successful surgical cure [5, 8, 13, 17–19, 21, 23–27]. For some institutions, MIRP has become the standard of care for the treatment of PHPT due to its improved success rates, reduced morbidity, increased patient satisfaction, and cost effectiveness when compared to traditional bilateral neck exploration [8, 21, 26, 28].

Serial intraoperative PTH (IOPTH) measurement has now been adopted by most institutions to gauge appropriate decrease in serum PTH levels, usually a 50% or greater decrease relative to preoperative values, following excision of an adenoma. Such a drop would indicate that the offending gland had successfully been removed [29]. IOPTH assessment has a high level of sensitivity (98%) and specificity (93%) for determining cure. However, a number of factors may contribute to the need for multiple unnecessary measurements, including timing and location of blood draw, variability in PTH half-life (particularly in patients with renal and/or liver failure), use of certain anesthetics such as propofol, and acute fluctuations in PTH levels secondary to physical manipulation of the adenoma itself, thereby further lengthening operative time [30]. Opportunities for further improvement in MIBI-avid parathyroid adenoma surgery remain, including minimizing time spent in the operating room (OR).

Real-time intraoperative imaging using a portable gamma camera can quickly confirm the absence or persistence of a MIBI-avid parathyroid adenoma within the surgical bed. A LFOVGC was developed recently as a lightweight, portable, general purpose planar imager by the Digirad Corporation (Ergo, Digirad Corporation, Poway, CA) allowing the opportunity to explore the utility of a LFOVGC for a variety of intraoperative uses, including parathyroid adenoma resection.

The purpose of this study was to demonstrate that intraoperative utilization of a LFOVGC can (1) accurately identify MIBI-avid parathyroid adenomas, (2) confirm complete resection of MIBI-avid parathyroid adenomas by imaging both the specimen and the neck, and (3) potentially significantly decrease the postresection “time-to-result” duration required for intraoperative confirmation of successful parathyroid adenoma removal.

## 2. Materials and Methods

Following approval by the Ohio State University Institutional Review Board, patients enrolled in our study were previously diagnosed with PHPT and had symptomatic hypercalcemia (serum total Ca > 10.0 mg/dL), elevated PTH (>72 pg/mL), and a single MIBI-avid parathyroid adenoma evident on preoperative MIBI imaging. Preoperative diagnostic imaging was performed either at the Ohio State University Wexner Medical Center (OSUWMC) (11/20 patients) or at an outside institution (9/20 patients). All diagnostic imaging was reviewed prior to surgery by an OSUWMC nuclear medicine physician and the surgeon to confirm scintigraphic findings compatible with abnormal MIBI-avid parathyroid tissue. Unless otherwise stated, patients underwent a minimally invasive parathyroidectomy (MIP), performed by either one of two surgeons at the OSUWMC. Adjuncts to surgery included intraoperative imaging utilizing a LFOVGC, serial IOPTH monitoring, and intraoperative pathologic examination of frozen sectioning.

**Figure 1:**
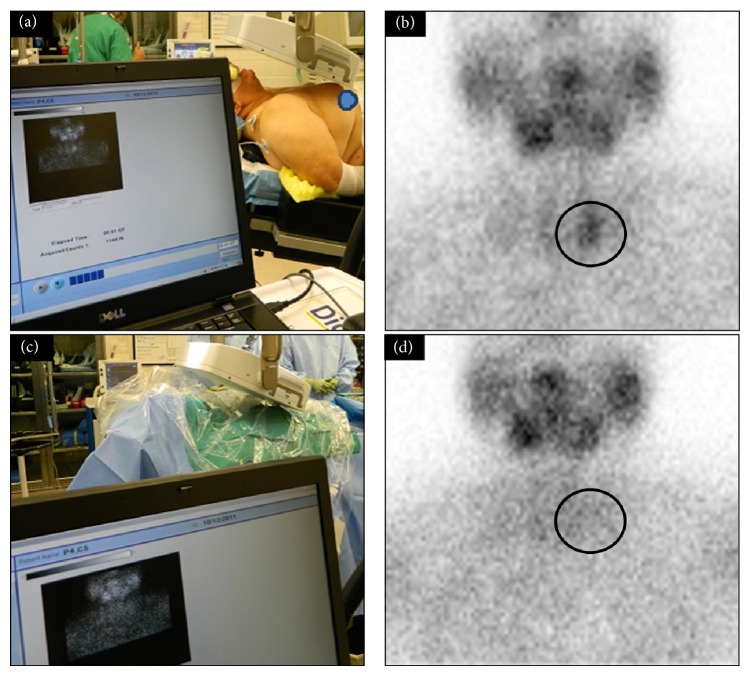
Digital photo demonstrating the intraoperative LFOVGC setup (a) for preincision intraoperative neck imaging. (b) A single focus of activity (black circle) in the left neck indicating a single parathyroid adenoma. Postexcision intraoperative LFOVGC setup with a sterile field is shown (c), as well as the related intraoperative postexcision image of the neck demonstrating no remaining activity (black circle) and complete removal of abnormal parathyroid tissue (d).

On the day of surgery, a dose of approximately 310 ± 8 MBq of MIBI was administered intravenously, 136 ± 29 minutes prior to surgery. A preoperative serum PTH level was obtained from the patient in the preoperative holding area. In the operating room, and prior to incision, the LFOVGC was used to image the patient's neck (Figure 1). The LOFVGC was used for imaging during radioguided surgery. Acquisition times of 5 minutes or less, for each image of the neck or specimen, were acquired and then were available for the surgeon to review in the operating room. The camera has a 15.6′′ × 12.2′′ field of view and is capable of detecting low energy gamma radiation from 50 to 350 KeV, which is appropriate for MIBI detection.

**Figure 2:**
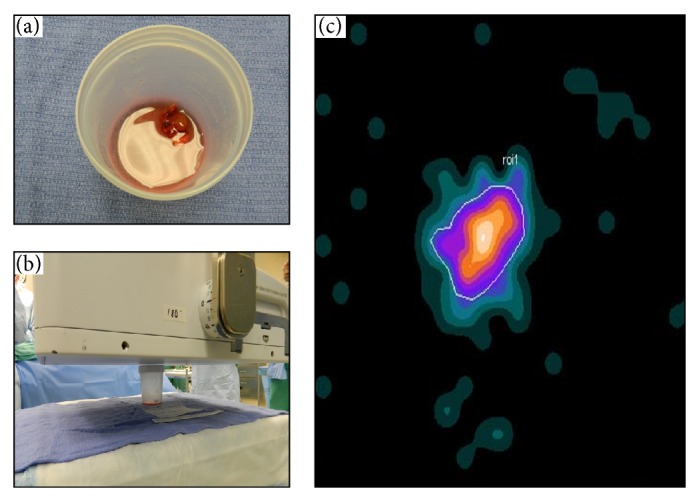
Digital photo of postexcision specimen (a), the intraoperative LFOVGC setup for imaging the specimen (b), and the resultant intraoperative LFOVGC image of a single MIBI-avid specimen, representing abnormal parathyroid tissue (c).

**Figure 3:**
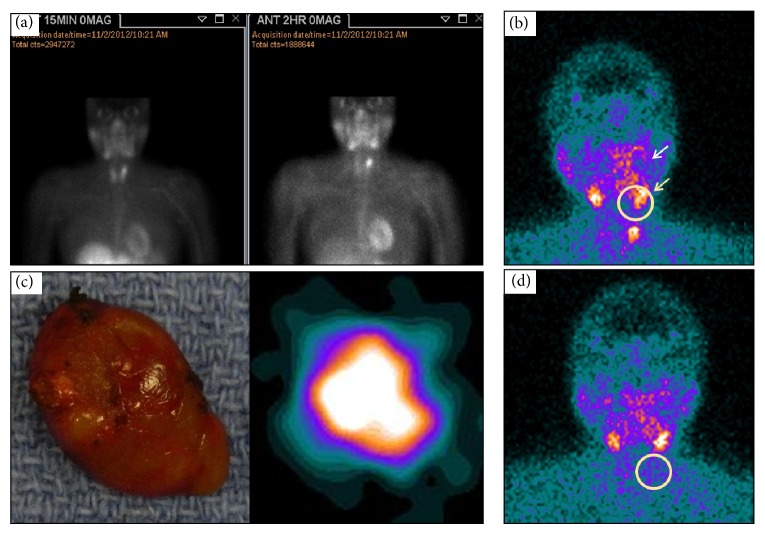
Preoperative MIBI planar image of a patient with positive diagnostic MIBI imaging at 15 minutes after injection (left image) and 120 minutes (right image) after administration of the radiolabeled agent showing activity in the left superior parathyroid (white arrow). (a) Intraoperative, preincision LFOVGC image identifying the single focus of activity (yellow circle) and normal uptake in the salivary glands (yellow arrow). (b) Digital photo of the resected tissue (left image) and the resultant LFOVGC image of the same excised tissue confirming MIBI-avid specimen (right image). (d) Intraoperative, postexcision, and LFOVGC image of the patient's neck showing absence of targeted residual activity (yellow circle) and verification of a complete resection.

The suspected location of the parathyroid adenoma was marked for reference. Immediately following surgical resection, the excised specimen was imaged with the LFOVGC to assess for presence of radiotracer uptake (Figure 2). The specimen was then sent to pathology for frozen section examination. Additionally, a postexcision image of the neck was taken to verify either absence of preoperative MIBI activity, indicating complete resection, or persistent/residual MIBI activity in the neck, indicating incomplete resection (Figure 3). In those instances where additional tissue was excised, all specimens were imaged and a final LFOVGC image of the neck was obtained for confirmation.

Concurrent with the acquisition of images, serial IOPTH measurements were obtained at 5 and/or 10 minutes after resection. If IOPTH levels remained more than 50% of preoperative values, additional PTH samples were obtained and the trend was assessed. Sterile technique was maintained for all equipment entering and exiting the surgical field. The times required for all neck and specimen imaging, as well as determined IOPTH and frozen section examination, were recorded and adjusted to OR time for comparison. All patients had preoperative and postoperative serum calcium and PTH measurements to verify normalization of their respective values and confirm cure.

*Statistics. Unless otherwise indicated, all values are expressed as mean ± standard error of the mean (SEM).*

## 3. Results

### 3.1. Patients

There were 20 patients diagnosed with sporadic PHPT enrolled consecutively to undergo surgery using the LFOVGC for intraoperative imaging. There were 4 males and 16 females with a mean age of 40 years (range 25–76) and 54 years (range 26–74), respectively. All patients had a single MIBI-avid adenoma evident on both preoperative diagnostic and intraoperative imaging. None of the patients were found to have ectopic or multiglandular disease at the time of surgery. All patients underwent a MIRP, with the exception of one patient who was also known to have a multinodular goiter and underwent a planned total thyroidectomy following parathyroidectomy (patient 14). Additionally, one patient had undergone a previous total thyroidectomy (patient 9); otherwise the remaining patients (19/20) had no history of previous neck surgery. Both patients (patients 14 and 9) were included in the study since surgery and the methods for detection and confirmation of removal of abnormal parathyroid tissue (IOPTH and intraoperative pathology) were carried out in a similar fashion for all 20 patients. The mean preoperative and postresection IOPTH values were 133 ± 39 pg/mL (range 70–212) and 28 ± 16 pg/mL (range 6–67), respectively. All patients underwent one surgical procedure with removal of one, two, or three specimens. All patients were cured of their hyperparathyroidism after this one exploration as demonstrated by normalization of their serum total calcium and PTH on the postoperative clinic visit 2 weeks later.

### 3.2. LFOVGC Imaging

The mean time from MIBI injection to preincision LFOVGC imaging was 136 ± 29 minutes (range 87–221). The length of time needed for completing intraoperative imaging is shown in Table 1. The total number of specimen and neck images was greater than 20 because more than one image was obtained per patient, if more than one specimen was resected. A postexcision neck image was not always performed if the initial specimen imaging (ES) was interpreted as negative and continued surgical exploration was performed. Therefore, the time interval between ES and postexcision neck imaging was variable. Postexcision neck imaging was not performed for all specimens removed and not immediately after specimen imaging in all cases where both images were obtained after resection.

### 3.3. Preincision LFOVGC Imaging

The mean time from preincision imaging to initial ES imaging was 47 ± 4 minutes (range 29–86; *n* = 19). One patient (patient 2) was excluded from this calculation because preincision image was acquired in the preoperative holding area and not in the operating room immediately prior to initiation of surgical procedure. The mean time from preincision imaging to initial postresection neck imaging was 51 ± 13 minutes (range 38–79; *n* = 16). The lag time between preincision imaging and specimen or postresection neck imaging did not affect image resolution or compromise the ability for interpretation of images.

### 3.4. Intraoperative LFOVGC Imaging

**Figure 4:**
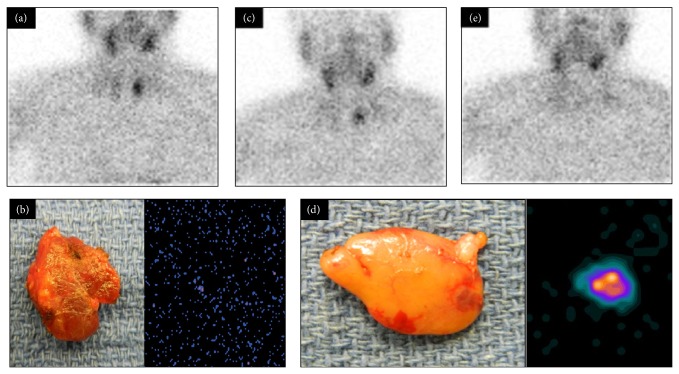
Example of pictorial presentation of planar imaging of a patient with positive diagnostic MIBI imaging (outside hospital) with positive same day preincision neck MIBI image (a), negative initial resected specimen LFOVGC MIBI image (b), and residual neck MIBI activity on first postexcision LFOVGC neck image (c). Resection of additional tissue as a result of these findings demonstrated MIBI-avid specimen LFOVGC image (d) and negative postexcision neck LFOVGC MIBI image (e).

There were 13/20 patients who had positive specimen imaging and negative postresection neck imaging upon initial resection, indicating complete surgical resection (Table 2). The remaining 7/20 patients went on to have two (5/7 patients) or three (2/7 patients) specimens resected during their surgery because of negative initial specimen imaging (5/7 patients) and/or postexcision neck imaging positive for residual radioactivity (3/7 patients) (Figure 4). In most of these cases the initial specimen removed was not parathyroid tissue (i.e., lymph node, fat, or thyroid tissue). One of these patients (patient 9) had both positive ES and postresection neck imaging, indicating incomplete adenoma resection. Both specimens were later confirmed on path to be histologically consistent with parathyroid adenoma tissue. There were no cases of multigland disease in any of the twenty patients studied.

### 3.5. Excised Specimen Imaging

**Figure 5:**
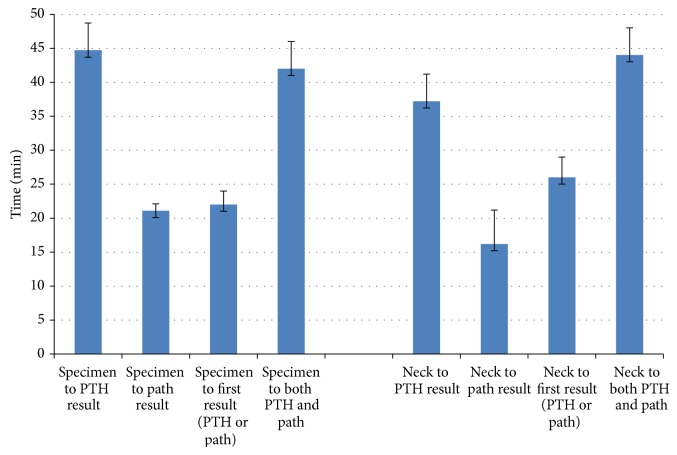
Graphic representation of time saving using specimen and neck imaging compared to path and/or PTH confirmation. Values represent mean of all specimens removed with error bars representing standard error of the mean. Specimen to PTH result: time from the beginning of specimen imaging to IOPTH results. Specimen to path result: time from the beginning of specimen imaging to time of pathology results. Specimen to first result (PTH or path): time from the start of specimen imaging to whichever result was reported first path or PTH. Specimen to both PTH and path: time from the beginning of specimen imaging until both PTH and path results were reported. Neck to PTH result: time from the beginning of neck imaging to IOPTH results. Neck to path result: time from the beginning of neck imaging to time of pathology results. Neck to first result (PTH or path): time from the start of neck imaging to whichever result was reported first path or PTH. Neck to both PTH and path: time from the beginning of neck imaging until both PTH and path results were reported.

The mean time from the beginning of specimen imaging (including imaging of positive and negative pathology specimens) to confirmation of intraoperative frozen section pathology analysis or IOPTH results was 21 ± 1 minutes (range 11–36; *n* = 26) and 45 ± 4 minutes (range 27–96; *n* = 23), respectively (Figure 5). Patient 1, specimen 1 imaging to path result, was excluded secondary to erroneous timestamp for path result in medical record. Four patients were excluded from the specimen imaging to PTH result secondary to no PTH sample being drawn (patient 12, specimen 1; patient 13, specimen 1; patient 17, specimen 1; patient 17, specimen 2).

The mean time from beginning of specimen imaging to receiving both frozen pathologic assessment and IOPTH results was 42.0 ± 4 minutes (range 24–96; *n* = 26). In one patient (patient 1, specimen 1), the frozen section results were significantly delayed and the true time is unknown. Therefore, time value for intraoperative pathologic reporting for this patient was excluded from the calculations.

The mean time from the beginning of specimen imaging to the reporting of either the pathologic assessment or IOPTH, whichever came first (first result), was 22 ± 2 minutes (range 11–44; *n* = 26) (Figure 5). One patient (patient 1, specimen 1) was excluded from this pathology calculation because the timestamp of lab results in the electronic medical record was erroneously delayed for unknown reasons.

### 3.6. Postresection Neck Imaging

The mean time from beginning of postresection neck imaging (including imaging of positive and negative pathology specimens) to confirmation of intraoperative frozen section pathology analysis or IOPTH results was 16 ± 5 minutes (range 7–26; *n* = 18) and 37 ± 4 minutes (range 12–91; *n* = 21), respectively (Figure 2). Four patients (patients 6, 12, 13, and 17) did not have any postresection neck imaging after first specimen removal. Four images were excluded for path confirmation and 1 image for IOPTH confirmation was excluded because imaging was delayed until after path or lab results were obtained, respectively (i.e., the procedure was completed and then the images were allowed to be obtained). One patient was excluded from this pathology calculation because the time of lab results in the electronic medical record was erroneously delayed by a significant amount.

The mean time from when postresection neck imaging was begun to when both frozen pathologic assessment and IOPTH results were reported was 44 ± 4 minutes (range 12–91; *n* = 22). In one patient, the frozen section results were significantly delayed and the true time is unknown. Therefore, time value for intraoperative pathologic reporting for this patient was excluded from the calculations. The mean time from the beginning of postresection neck imaging to the reporting of either the pathologic assessment or IOPTH, whichever came first (first result), was 26 ± 3 minutes (range 7–79; *n* = 23) (Figure 5). One patient (patient 1, specimen 1) was excluded from this pathology calculation because the timestamp of lab results in the electronic medical record was erroneously delayed for unknown reasons.

In many instances we found that either the specimen or postexcision neck imaging result, more commonly the ES specimen, led to foregoing the corresponding paired postexcision neck image or the specimen image. Other times the paired image (more commonly the neck) was acquired significantly later during the process because the ES result was known. Therefore, the time from ES imaging to results was significantly shorter than postexcision neck imaging to results, in many cases. Additionally, delays in postexcision neck imaging also resulted in negative values for calculations comparing the postexcision neck imaging with either path calculations (patient 2, specimen 2; patient 6, specimen 2) or IOPTH (patient 1, specimen 1). These values were excluded from the calculations.

Qualitative evaluation of the intraoperative neck and specimen imaging, for the presence or absence of activity to confirm abnormal parathyroid tissue, yielded a 100% correlation between intraoperative imaging, IOPTH results, and final pathologic examination (Table 3).

## 4. Discussion

This is the first study, to our knowledge, where a LFOVGC was used for intraoperative assessment of the preoperative field, resected specimen, and the postresection surgical field to evaluate completeness of resection in parathyroid disease. Our results demonstrate for the first time that LFOVGC is capable of providing an accurate preoperative image guiding a focused approach to removal and, more importantly, that it provided useful postresection images of both the excised specimen and neck to confirm adequate resection or direct subsequent reresection, if needed. The LFOVGC was a useful adjunct to parathyroidectomy for MIBI-avid disease in patients with PHPT. Additionally, qualitative evaluation of intraoperative neck and specimen imaging was 100% sensitive and 100% specific for detecting MIBI-avid parathyroid adenomas.

Surgical management for PHPT has evolved towards minimally invasive surgery by using a small, unilateral neck incision in order to improve cosmesis, decrease the likelihood of surgical complications and morbidity, and shorten the length of time spent in the operating room. This has been accomplished, in part, with the combined use of preoperative parathyroid scintigraphy, intraoperative gamma detection probe, and verification of completeness of resection with serial IOPTH measurement and intraoperative pathologic frozen section evaluation of the resected tissue. Recent studies have shown that these techniques can achieve equivalent cure rates to that of formal bilateral neck exploration [8, 21, 26, 28, 31].

The use of IOPTH for predicting surgical cure after parathyroidectomy is well established and has become the standard of care in verifying completeness of surgical resection after parathyroidectomy [18, 26, 32–36]. Moreover, IOPTH is a valuable tool to reliably predict surgical cure in patients with single gland and multigland disease [37]. However, some authors argue that IOPTH may not be absolutely necessary given the accuracy of MIRP [15] and routine use of IOPTH may increase cost appreciably while marginally increasing cure rate [38]. The incidence of solitary adenoma was historically accepted to be 80%, a figure largely based on a 1981 study by Bruining et al., after investigating the pathology of 615 patients treated for PHPT [39]. Therefore, the theoretical failure rate for MIBI imaging was presumed to be at least 20% in all patients with PHPT. However, Denham et al. found the true incidence of solitary adenoma to be closer to 87%, with hyperplasia accounting for approximately 9% and multiple-gland disease approximately 3% based on a large systematic review of 6,331 patients with PHPT [2, 39]. Furthermore, adenomas typically contain at least 20% or more of background radioactivity, compared to hyperplastic glands which usually do not retain activity. Thus, adenomas are more distinguishable on MIBI imaging [40]. Even though MIBI diagnostic preoperative imaging is not 100% sensitive for identifying all single gland disease, the failure rates are more likely low.

Additionally, MIRP has also been reported to have a positive predictive value of approximately 97% for detecting MIBI-avid parathyroid adenomas in patients with single gland disease [41]. Denham reported that, for patients with a positive MIBI scan, the failure rate of the surgery after removing the gland was less than 1%. However, one study reported that 27% percent of the time gamma readings demonstrated lower indices, which lead to extended surgeries in search of multiglandular disease [27]. Despite improvements in reducing overall surgical morbidity, there still remains a significant intraoperative time commitment when taking into consideration the length of time needed for confirming intraoperative PTH and pathology results.

Therefore, we embarked on this pilot study to evaluate the potential for intraoperative gamma imaging, utilizing a LFOVGC, for verification of completeness of surgical resection, and determine the time it required. Our results showed a direct correlation for both ex vivo specimen imaging and postresection neck imaging with respect to IOPTH results and the final pathology determination. If the excised specimen showed no evidence of radioactivity or there was residual radioactivity in the neck on postexcision imaging, it was confirmed to have been a negative specimen on pathologic examination in all cases. Reciprocally, in those patients with positive specimen and negative neck imaging, the pathology confirmed successful removal of the involved adenoma in all cases.

Our results support the contention that obtaining a real-time complete anatomic image of the neck and upper chest can facilitate accurate adenoma localization and confirmation of resection. This technique could also eliminate the potential for inaccurate interpretation of nonspecific background radioactivity often encountered with the use of HGDP for evaluating pathologic glands. Though not examined in this study, the LFOVGC can easily be used to obtain lateral views of the neck in addition to views of the anterior neck; thereby, providing 2-dimensional viewing of the entire neck may be advantageous in cases of ectopic disease. On average, using ex vivo specimen imaging as the determinant of whether the tissue removed was abnormal parathyroid tissue would have translated to a mean time saving advantage of 42 minutes compared to verification by both frozen section pathology and postresection IOPTH results or 22 minutes if the procedure was considered successful when either the frozen section pathology or IOPTH results were reported (whichever was reported first).

The utility of portable gamma cameras for intraoperative imaging has been assessed by only a few investigators, and though these studies showed similar efficacy to that of our results using a LFOVGC, the potential benefits of specimen imaging and estimation of a time saving potential were not examined [42–46].

Our results demonstrate the use of intraoperative imaging with a LFOVGC not only can accurately verify completeness of surgical resection but also is more expedient than IOPTH and pathologic confirmation, thereby decreasing operative time if used alone. Despite the fact that IOPTH and intraoperative frozen sectioning has a 95% success rate [5, 7, 8] for determining surgical cure this could be further improved with use of a radioguided probe [41] and significantly reduced time in the OR.

We acknowledge the limitations of this pilot study, including a relatively small sample size and the lack of a control group for comparison or randomization. Additionally, not all patients underwent both ex vivo specimen imaging and postexcision neck imaging after removal of each individual specimen. Given the ease of interpretation of the initial images, the need for paired ES/neck image after every single resection was quickly deemed unnecessary by the surgeon. Further validation of our findings with a prospective, randomized clinical trial is warranted to verify accuracy and efficacy with use of the LFOVGC for intraoperative imaging.

## 5. Conclusion

This initial study demonstrates that real-time intraoperative gamma camera imaging using a LFOVGC has the ability to accurately identify MIBI-avid parathyroid adenomas. Additionally, it has the ability to confirm complete resection of MIBI-avid parathyroid adenomas in real-time via both ES and neck imaging. In these patients, we found that LFOVGC was able to efficiently predict complete resection of MIBI-avid hyperfunctioning parathyroid tissue for patients undergoing parathyroidectomy for PHPT. The LFOVGC accurately predicted the end of the operation an average of 25 to 30 minutes faster than the traditional IOPTH and path methods. Clearly, larger studies are needed to verify these initial results, but LFOVGC may prove to be a useful adjunct for the parathyroid surgeon.

## Figures and Tables

**Table 1:** Distribution of duration of image acquisition for preincision neck imaging, postexcision specimen imaging, and postexcision neck imaging.

	Time				
	1 minute	2 minutes	3 minutes	5 minutes	Total
Preincision neck	0	7	12	1	20
Specimen	28	0	2	0	30
Postexcision neck	1	8	14	2	25

**Table 2:** Patient details on positivity/negativity of excised specimen (ES) and neck LFOVGC imaging. Details per patient include number of intraoperative specimen and neck LFOVGC images and whether each image was positive or negative for evidence of MIBI-avid abnormal parathyroid tissue.

Patient	Specimen imaging results			
	1st	2nd	3rd	Total OR time (hour:minute)
	ES/neck	Spec./postexc.	Spec./postexc.	
1	+/−	X	X	1:17
2	NP/+	+/−	X	2:00
3	+/−	X	X	1:10
4	+/−	X	X	1:05
5	+/−	X	X	1:41
6	−/NP	+/−	X	2:19
7	+/−	X	X	1:36
8	+/−	X	X	1:15
9	+/+	+/−	X	1:23
10	+/−	X	X	1:41
11	+/−	X	X	1:15
12	−/NP	+/−	X	1:32
13	−/NP	−/NP	+/−	2:26
14	−/+	+/−	X	2:25
15	+/−	X	X	2:01
16	+/−	X	X	1:08
17	−/NP	NP/NP	+/−	2:08
18	+/−	X	X	1:19
19	+/−	X	X	1:13
20	+/−	X	X	1:12

X: not applicable.

+: positive imaging.

−: negative imaging.

NP: not performed.

**Table 3:** Contingency table of imaging findings versus pathology findings for excised specimens and neck imaging.

Specimen imaging versus pathology			Postexcision neck imaging versus pathology		
	Pathology positive	Pathology negative		Pathology positive	Pathology negative
Specimen imaging positive	20	0	Neck imaging residual focus	20	0
Specimen imaging negative	0	7	Neck imaging focus gone	0	7

True positives for specimen imaging (specimen hot adenoma removed)					=100%
True positives for neck imaging (focus present adenoma not removed)					=100%
False positives for specimen imaging (specimen hot adenoma not removed)					=0%
False positives for neck imaging (focus present adenoma removed)					=0%
True negatives for specimen imaging (specimen cold adenoma removed)					=100%
True negatives for neck imaging (focus absent adenoma removed)					=100%
False negatives for specimen imaging (specimen cold adenoma not removed)					=0%
False negatives for neck imaging (focus absent adenoma not removed)					=0%

## References

[1] Marcocci C., Cetani F. (2011). Primary hyperparathyroidism. *The New England Journal of Medicine*.

[2] Denham D. W., Norman J. (1998). Cost-effectiveness of preoperative sestamibi scan for primary hyperparathyroidism is dependent solely upon the surgeon's choice of operative procedure. *Journal of the American College of Surgeons*.

[3] van Heerden J. A., Grant C. S. (1991). Surgical treatment of primary hyperparathyroidism: an institutional perspective. *World Journal of Surgery*.

[4] Kaplan E. L., Yashiro T., Salti G. (1992). Primary hyperparathyroidism in the 1990s: choice of surgical procedures for this disease. *Annals of Surgery*.

[5] Morrow J. S., Miller R. H. (1994). Diagnosis and management of primary hyperparathyroidism. *The Journal of the Louisiana State Medical Society*.

[6] Cappello Z. J., Bumpous J. M. (2013). Is bilateral exploration still the standard of care for primary hyperparathyroidism?: outcomes of focused radio-guided parathyroidectomy and bilateral explorations. *Laryngoscope*.

[7] Chen H., Zeiger M. A., Gordon T. A. (1996). Parathyroidectomy in Maryland: effects of an endocrine center. *Surgery*.

[8] Udelsman R., Lin Z., Donovan P. (2011). The superiority of minimally invasive parathyroidectomy based on 1650 consecutive patients with primary hyperparathyroidism. *Annals of Surgery*.

[9] Ammori B. J., Madan M., Gopichandran T. D. (1998). Ultrasound-guided unilateral neck exploration for sporadic primary hyperparathyroidism: is it worthwhile?. *Annals of the Royal College of Surgeons of England*.

[10] Bozkurt M. F., Uğur Ö., Hamaloğlu E., Sayek I., Gulec S. A. (2003). Optimization of the gamma probe-guided parathyroidectomy. *The American Surgeon*.

[11] Gogas J., Kouskos E., Mantas D. (2003). Pre-operative Tc-99m-sestamibi scanning and intra-operative nuclear mapping: are they accurate in localizing parathyroid adenomas?. *Acta Chirurgica Belgica*.

[12] Vassy W. M., Nelson H. S., Mancini M. L., Timaran C. H., Hall N. C., Smith G. T. (2003). Minimally invasive parathyroidectomy: how effective is preoperative sestamibi scanning?. *The American Surgeon*.

[13] Kell M. R., Sweeney K. J., Moran C. J., Flanagan F., Kerin M. J., Gorey T. F. (2004). Minimally invasive parathyroidectomy with operative ultrasound localization of the adenoma. *Surgical Endoscopy and Other Interventional Techniques*.

[14] Lee W.-J., Ruda J., Stack B. C. (2004). Minimally invasive radioguided parathyroidectomy using intraoperative sestamibi localization. *Otolaryngologic Clinics of North America*.

[15] Caudle A. S., Brier S. E., Calvo B. F., Hong J. K., Meyers M. O., Ollila D. W. (2006). Experienced radio-guided surgery teams can successfully perform minimally invasive radio-guided parathyroidectomy without intraoperative parathyroid hormone assays. *The American Surgeon*.

[16] Ollila D. W., Caudle A. S., Cance W. G. (2006). Successful minimally invasive parathyroidectomy for primary hyperparathyroidism without using intraoperative parathyroid hormone assays. *The American Journal of Surgery*.

[17] Davis M. L., Quayle F. J., Middleton W. D. (2007). Ultrasound facilitates minimally invasive parathyroidectomy in patients lacking definitive localization from preoperative sestamibi scan. *The American Journal of Surgery*.

[18] Fraker D. L., Harsono H., Lewis R. (2009). Minimally invasive parathyroidectomy: benefits and requirements of localization, diagnosis, and intraoperative PTH monitoring. long-term results. *World Journal of Surgery*.

[19] Haciyanli M., Genc H., Damburaci N., Oruk G., Tutuncuoglu P., Erdogan N. (2009). Minimally invasive focused parathyroidectomy without using intraoperative parathyroid hormone monitoring or gamma probe. *Journal of Postgraduate Medicine*.

[20] Flynn M. B., Civelek A. C. (2010). Current status of surgical techniques for parathyroidectomy for untreated primary hyperparathyroidism: is the technology worth it?. *The American Surgeon*.

[21] Ikeda Y., Takayama J., Takami H. (2010). Minimally invasive radioguided parathyroidectomy for hyperparathyroidism. *Annals of Nuclear Medicine*.

[22] Martinez D. A., King D. R., Romshe C. (1995). Intraoperative identification of parathyroid gland pathology: a new approach. *Journal of Pediatric Surgery*.

[23] Haim M. B., Zwas S. T., Munz Y. (2003). Focused, minimally invasive radio-guided parathyroidectomy: a feasible and safe option for elderly patients with primary hyperparathyroidism. *Israel Medical Association Journal*.

[24] Fuster D., Ybarra J., Ortin J. (2006). Role of pre-operative imaging using ^99m^Tc-MIBI and neck ultrasound in patients with secondary hyperparathyroidism who are candidates for subtotal parathyroidectomy. *European Journal of Nuclear Medicine and Molecular Imaging*.

[25] Soon P. S., Delbridge L. W., Sywak M. S., Barraclough B. M., Edhouse P., Sidhu S. B. (2008). Surgeon performed ultrasound facilitates minimally invasive parathyroidectomy by the focused lateral mini-incision approach. *World Journal of Surgery*.

[26] Adil E., Adil T., Fedok F., Kauffman G., Goldenberg D. (2009). Minimally invasive radioguided parathyroidectomy performed for primary hyperparathyroidism. *Otolaryngology—Head and Neck Surgery*.

[27] García-Talavera P., García-Talavera J.-R., González C., Martín E., Martín M., Gómez A. (2011). Efficacy of in-vivo counting in parathyroid radioguided surgery and usefulness of its association with scintigraphy and intraoperative PTHi. *Nuclear Medicine Communications*.

[28] Zanocco K., Heller M., Sturgeon C. (2011). Cost-effectiveness of parathyroidectomy for primary hyperparathyroidism. *Endocrine Practice*.

[29] Inabnet W. B. (2004). Intraoperative parathyroid hormone monitoring. *World Journal of Surgery*.

[30] Richards M. L., Grant C. S. (2007). Current applications of the intraoperative parathyroid hormone assay in parathyroid surgery. *The American Surgeon*.

[31] Hughes D. T., Miller B. S., Park P. B., Cohen M. S., Doherty G. M., Gauger P. G. (2013). Factors in conversion from minimally invasive parathyroidectomy to bilateral parathyroid exploration for primary hyperparathyroidism. *Surgery*.

[32] Di Stasio E., Carrozza C., Lombardi C. P. (2007). Parathyroidectomy monitored by intra-operative PTH: the relevance of the 20 min end-point. *Clinical Biochemistry*.

[33] Irvin G. L., Molinari A. S., Figueroa C., Carneiro D. M. (1999). Improved success rate in reoperative parathyroidectomy with intraoperative PTH assay. *Annals of Surgery*.

[34] Seybt M. W., Loftus K. A., Mulloy A. L., Terris D. J. (2009). Optimal use of intraoperative PTH levels in parathyroidectomy. *Laryngoscope*.

[35] Woodrum D. T., Saunders B. D., England B. G., Burney R. E., Doherty G. M., Gauger P. G. (2004). The influence of sample site on intraoperative PTH monitoring during parathyroidectomy. *Surgery*.

[36] Ypsilantis E., Charfare H., Wassif W. S. (2010). Intraoperative PTH assay during minimally invasive parathyroidectomy may be helpful in the detection of double adenomas and may minimise the risk of recurrent surgery. *International Journal of Endocrinology*.

[37] Hughes D. T., Miller B. S., Doherty G. M., Gauger P. G. (2011). Intraoperative parathyroid hormone monitoring in patients with recognized multiglandular primary hyperparathyroidism. *World Journal of Surgery*.

[38] Morris L. F., Zanocco K., Ituarte P. H. (2010). The value of intraoperative parathyroid hormone monitoring in localized primary hyperparathyroidism: a cost analysis. *Annals of Surgical Oncology*.

[39] Bruining H. A., van Houten H., Juttmann J. R., Lamberts S. W. J., Birkenhäger J. C. (1981). Results of operative treatment of 615 patients with primary hyperparathyroidism. *World Journal of Surgery*.

[40] Norman J., Politz D. (2009). 5,000 parathyroid operations without frozen section or PTH assays: measuring individual parathyroid gland hormone production in real time. *Annals of Surgical Oncology*.

[41] Shabtai M., Ben-Haim M., Muntz Y. (2003). 140 Consecutive cases of minimally invasive, radio-guided parathyroidectomy: lessons learned and long-term results. *Surgical Endoscopy and Other Interventional Techniques*.

[42] Cassinello N., Ortega J., Lledo S. (2009). Intraoperative real-time ^99m^Tc-sestamibi scintigraphy with miniature gamma camera allows minimally invasive parathyroidectomy without ioPTH determination in primary hyperparathyroidism. *Langenbeck's Archives of Surgery*.

[43] Fujii T., Yamaguchi S., Yajima R. (2011). Use of a handheld, semiconductor (cadmium zinc telluride)-based gamma camera in navigation surgery for primary hyperparathyroidism. *The American Surgeon*.

[44] Kitagawa W., Shimizu K., Akasu H. (2003). Radioguided parathyroidectomy for primary hyperparathyroidism using the solid-state, multi-crystal gamma camera. *Medical Science Monitor*.

[45] Ortega J., Ferrer-Rebolleda J., Cassinello N., Lledo S. (2007). Potential role of a new hand-held miniature gamma camera in performing minimally invasive parathyroidectomy. *European Journal of Nuclear Medicine and Molecular Imaging*.

[46] Rahbar K., Colombo-Benkmann M., Haane C. (2012). Intraoperative 3-D mapping of parathyroid adenoma using freehand SPECT. *EJNMMI Research*.

